# The dose-response association between estimated glomerular filtration rate and prognosis of patients with ST-segment elevation myocardial infarction from rural areas of China's Liaoning province: Erratum

**DOI:** 10.1097/MD.0000000000010259

**Published:** 2018-03-23

In the article, “The dose-response association between estimated glomerular filtration rate and prognosis of patients with ST-segment elevation myocardial infarction from rural areas of China's Liaoning province”,^[1]^ which appeared in Volume 96, Issue 52 of *Medicine*, affiliations a and b should have appeared as:

a. Department of Clinical Epidemiology, Institute of Cardiovascular Diseases, First Affiliated Hospital of China Medical University, Shenyang, China.
b. Department of Geriatric Cardiology, First Affiliated Hospital of China Medical University, Shenyang, China.
